# Differential Apoptotic Effects of Bee Product Mixtures on Normal and Cancer Hepatic Cells

**DOI:** 10.3390/antiox12030615

**Published:** 2023-03-02

**Authors:** Vanesa Sánchez-Martín, Paloma Morales, Amaia Iriondo-DeHond, Xavier F. Hospital, Manuela Fernández, Eva Hierro, Ana I. Haza

**Affiliations:** 1 Departamento de Nutrición y Ciencia de los Alimentos, Sección Departamental de Nutrición y Ciencia de los Alimentos, Facultad de Veterinaria, Universidad Complutense, 28040 Madrid, Spain; 2 Departamento de Farmacia Galénica y Tecnología de los Alimentos, Sección Departamental de Farmacia Galénica y Tecnología de los Alimentos, Facultad de Veterinaria, Universidad Complutense, 28040 Madrid, Spain

**Keywords:** antioxidant honey mixtures, apoptosis, bee products, cancer, cytotoxicity, honey, propolis, royal jelly

## Abstract

Most effective anticancer drugs normally generate considerable cytotoxicity in normal cells; therefore, the preferential activation of apoptosis in cancer cells and the reduction of toxicity in normal cells is a great challenge in cancer research. Natural products with selective anticancer properties used as complementary medicine can help to achieve this goal. The aim of the present study was to analyze the effect of the addition of bee products [propolis (PR) or royal jelly (RJ) or propolis and royal jelly (PR+RJ), 2–10%] to thyme (TH) and chestnut honeys (CH) on the differential anticancer properties, mainly the cytotoxic and pro-apoptotic effects, in normal and cancer hepatic cells. The cytotoxic effects of samples were analyzed using the MTT (3-(4,5-dimethylthiazol-2-yl)-2,5-diphenyltetrazolium bromide) assay (0–250 mg/mL) and the effects on apoptosis were analyzed using cell cycle analysis, TdT-dUTP terminal nick-end labeling (TUNEL) assay, DR5 (Death Receptor 5) and BAX (BCL-2-Associated X) activation, and caspases 8, 9, and 3 activities. Both honey samples alone and honey mixtures had no or very little apoptotic effect on normal cells. Antioxidant honey mixtures enhanced the apoptotic capacity of the corresponding honey alone via both extrinsic and intrinsic pathways. Of all the samples, chestnut honey enriched with 10% royal jelly and 10% propolis (sample 14, CH+10RJ+10PR) showed the highest apoptotic effect on tumor liver cells. The enrichment of monofloral honey with bee products could be used together with conventional anticancer treatments as a dietary supplement without side effects. On the other hand, it could be included in the diet as a natural sweetener with high added value.

## 1. Introduction

Bees are critically important to the environment since they enrich human life and can contribute to achieving the Sustainable Development Goals (SDG) proposed by United Nations. They promote biodiversity (goal 15), provide decent jobs (goal 8), and fight poverty (goal 1) and hunger (goal 2) [1]. A threat to bees is a threat to global food security. According to the Food and Agriculture Organization of the United Nations (FAO), of the 100 crop species that provide 90% of food worldwide, 71 are pollinated by bees. In the last 15 years, an unusual weakening of bee numbers was reported in Spain, among other Western European countries, due to climate change, intensive agriculture and pesticide use, starvation and poor bee nutrition, viruses, and attacks by pathogens and invasive species [2,3,4]. One of the many ways to save the bees is to support local beekeepers by consuming local honey and buying bee-derived products [5].

Bee products have long been used in traditional medicine, but they have recently gained interest from modern consumers due to the presence of phytochemicals with health-promoting properties, such as antioxidant, wound healing, and anti-inflammatory and anticancer activities [3,6]. Bee product consumption is considered a type of complementary medicine that is widely used among cancer patients with the potential to improve outcomes by helping patients tolerate conventional medical care and complete their recommended therapy [7]. However, most effective anticancer drugs normally generate considerable cytotoxicity in normal cells, which limits their clinical applications [8].

Natural products rich in antioxidants, such as berries, were proposed to support colon cancer chemoprevention or chemotherapy due to their anticancer properties [9]. In this context, honey has the potential to be used together with conventional cancer treatment to increase the anticancer effects of chemotherapy and to reduce its cytotoxic side effects [10,11,12,13]. In addition, data obtained from randomized clinical trials indicate that besides honey, the use of other bee products, such as propolis and royal jelly, seems to be very appropriate for the prevention and treatment of oral mucositis induced by radiotherapy, chemotherapy, or a combination of both [14].

The regulation of apoptosis is critical in cancer pathogenesis, as a failure to undergo apoptosis results in an uncontrolled increase in cancerous cells [15]. Honey was shown to induce apoptosis in numerous forms of tumor cells, making it a potential natural anticancer agent [15]. In this way, manuka honey induces apoptosis in epithelial cancer cells, and *Quercus pyrenaica* honeydew honey, with its high phenolic content, causes DNA damage and apoptosis in gastric adenocarcinoma cells [16,17]. Anticancer properties via apoptosis induction of cancer cells were also described for propolis and royal jelly [18,19]. Chinese propolis was shown to induce apoptosis and inhibit the proliferation of human gastric cancer cells [20]. In addition to inducing apoptosis, propolis suppresses tumor angiogenesis in vivo [21]. Royal jelly treatment was demonstrated to reduce the development of breast tumors in mice, and royal jelly proteins reduced the development of leukemia [22,23].

Bioactive compounds from natural sources have the potential to synergistically function as multi-target drugs at low doses that act through multiple mechanisms or signaling pathways for cancer treatment [24,25]. It was described that natural product mixtures are often more effective than purified compounds due to beneficial synergistic interactions [26]. For instance, vitamin C in combination with organosulfur compounds or with isothiocianates was a stronger inhibitor of induced oxidative DNA damage than each of these compounds alone [27]. On the other hand, a mixture containing quercetin, curcumin, green tea, cruciferex, and resveratrol demonstrated significant inhibition of the growth of squamous cell carcinoma and a dose-dependent inhibition of cell proliferation, matrix metalloproteinase (MMP)-2 and -9 secretion, and cell migration and invasion [28]. In addition, the combination of cordycepin, resveratrol, and genistein produced significant synergistic anticancer activities through different apoptosis-related pathways [25].

However, there is a lack of information on the effect of bee product mixtures (honey enriched with propolis and royal jelly) on apoptosis induction to support cancer treatment and avoid the development of chemotherapy side effects. In fact, monofloral honey samples enriched with propolis or propolis and royal jelly showed greater antioxidant properties and increased protective effects against induced DNA damage [29,30]. The preferential activation of apoptosis in cancer cells and the reduction of toxicity in normal cells is a great challenge in cancer research [8]. Consequently, the aim of the present study was to analyze the effects of the addition of bee products (propolis or royal jelly or propolis and royal jelly, 2–10%) to thyme and chestnut honeys on the differential anticancer properties, mainly the cytotoxic and pro-apoptotic effects, in normal and cancer hepatic cells.

## 2. Materials and Methods

### 2.1. Chemicals

MTT (3-(4,5-dimethylthiazol-2-yl)-2,5-diphenyl-tetrazolium bromide) (Cell Proliferation Kit I) and the In Situ Cell Death Detection Kit, Fluorescein (11684795910), were purchased from Roche (Indianapolis, IN, USA). Hoechst 33,342 was from Invitrogen, Thermo Fisher Scientific (Waltham, MA, USA). Etoposide, propidium iodide, RNase A, CHAPS (3-((3-Cholamidopropyl)dimethylammonio)-1-propanesulfonate hydrate), Protease Inhibitor Cocktail Set I (Calbiochem) and ethidium bromide were obtained from Sigma-Aldrich (St. Louis, MO, USA). Fluorogenic substrates of caspases 3, 8, and 9 were purchased from Enzo Life Sciences (Farmingdale, NY, USA). FITC Anti-DR5 (Death Receptor 5) antibody (DR5-01-1, ab53319) and FITC Anti-BAX (BCL-2 Associated X) antibody (T22-A, ab139543) were purchased from Abcam (Cambridge, UK). Dulbecco’s Modified Eagle Medium (DMEM), fetal calf serum, penicillin and streptomycin, and L-glutamine were purchased from Gibco Laboratories (Life Technologies, Inc., Gaithersburg, MD 20884-9980, USA).

### 2.2. Samples

Two monofloral honey samples, namely, sample 1 (thyme honey, TH) and sample 6 (chestnut honey, CH), were obtained in February–March 2021 directly from beekeepers in each region. Table 1 shows the scientific and common names, types, families, and geographic regions of thyme and chestnut honey samples, royal jelly (RJ), and propolis (PR). The analysis of the physicochemical parameters of both honey samples showed values within the limits established by the Spanish Royal Decree Law (RD) 1049/2003 [29,31]. The thyme and chestnut honey samples were mixed with royal jelly (RJ) and propolis (PR) (2–10%) to obtain the 17 samples analyzed in this study (Table 2).

Samples were prepared as described in Sánchez-Martín et al. [29]. Briefly, samples 1–17 were weighed and diluted in sterile distilled water at a final concentration of 1 g/mL, except for sample number 15 (RJ), which was prepared at 0.5 g/mL. Artificial honey (AH) was prepared by diluting 4.05 g of fructose, 3.35 g of glucose, 0.15 g of sucrose, and 0.75 g of maltose in sterile distilled water (1 g/mL). Then, samples were filtered (0.45 µm) and stored at −20 °C.

### 2.3. Cell Culture

HepG2 cells, from hepatocellular carcinoma, were provided by the Unidad de Terapias Farmacológicas and Unidad de Genética Molecular (Instituto de Investigación de Enfermedades Raras, Instituto de Salud Carlos III, Madrid, Spain), and WRL-68 cells, i.e., normal human liver cells, were obtained from the Centro de Instrumentación Científica (CIC) of the Universidad de Granada, Spain. Both cell lines were cultured in Dulbecco’s modified Eagle’s medium supplemented with 10% *v/v* fetal bovine serum (heat-inactivated), 50 µg/mL streptomycin, 50 U/mL penicillin, and 50 µg/mL L-glutamine. Cells were incubated at 37 °C and 100% humidity in a 5% CO_2_ atmosphere.

### 2.4. MTT (3-(4,5-dimethylthiazol-2-yl)-2,5-diphenyl-tetrazolium bromide) Assay

The effects of the samples on cell viability were determined using the MTT assay. HepG2 and WRL-68 cells were cultured at a density of 1 × 10^5^ cells per well of a 96-well plate for 24 h. Then, cells were treated with honey and bee product samples (Table 2) at concentrations of 0–250 mg/mL for 24, 48, and 72 h. After incubation, 10 µL of MTT Labeling Reagent was added to each well and incubated for 4 h at 37 °C, and then 100 μL of solubilization solution was added to each well. After 24 h, the optical density of each well was read at 620 nm (test wavelength) and 690 nm (reference wavelength) using a microplate reader (iEMS Reader MF, Labsystems, Helsinki, Finland). Experiments were carried out in triplicate (n = 16). Results were expressed as the percentage of viability (% cell survival) compared with the negative control (non-treated cells).

### 2.5. Cell Cycle Analysis

The cell cycle consists of several phases that depend on the DNA content of the cells, and the apoptotic cell population is located in the sub-G1 phase (fragmented DNA) [32]. Cell cycle analysis was assessed using flow cytometry after staining with propidium iodide, as described by Sánchez-Martín et al. [33]. First, 1 × 10^6^ HepG2 or WRL-68 cells per well of a 6-well plate were seeded. After 24 h, cells were treated with the different samples (Table 2) at 0–100 mg/mL for 24, 48, and 72 h. Non-treated cells were used as a negative control and cells treated with etoposide 50 µM were used as a positive control. Then, cells were detached with trypsin, washed twice with PBS 1X, and fixed in 70% ethanol. DNA was stained with 50 μg/mL propidium iodide and RNase A (1 mg/mL) for 30 min at 37 °C in a dark environment. Finally, fluorescence was measured using a FACS Calibur flow cytometer (Beckton Dickinson, Franklin Lakes, NJ, USA) from the Flow Cytometry and Fluorescence Microscopy Unit of the Universidad Complutense of Madrid (UCM) and Flowing Software 2 (University of Turku, Finland). Results were expressed as the percentage of cells in the sub-G1 phase over the total cells.

### 2.6. TdT-dUTP Terminal Nick-End Labeling (TUNEL) Assay

The TUNEL assay is used to detect DNA degradation, which occurs during the last stages of the apoptotic process [34]. Apoptotic cell death was measured using the In Situ Cell Death Detection Kit, Fluorescein, according to the manufacturer’s instructions. First, 1 × 10^6^ HepG2 or WRL-68 cells per well of a 6-well plate were seeded. After 24 h, cells were treated with the different samples (Table 2) at 0–100 mg/mL for 24, 48, and 72 h. Non-treated cells were used as a negative control and cells treated with etoposide 50 µM were used as a positive control. Then, cells were detached with trypsin, counted, and a suspension of 1 × 10^6^ cells was washed with PBS 1X and fixed in 2% formaldehyde in PBS (100 µL) for 1 h at room temperature with gentle shaking. Cells were permeabilized with 0.1% Triton X-100 in 0.1% sodium citrate for 2 min on ice, washed twice with PBS 1X, and incubated with the TUNEL reaction mixture (50 μL of enzyme solution (TdT) and 450 μL of label solution (fluoresceindUTP)) for 1 h at 37 °C in the dark in a humidified atmosphere. The omission of TdT from the staining protocol constituted the negative control. Finally, the percentage of apoptotic cells was measured using a FACS Calibur flow cytometer (Beckton Dickinson, Franklin Lakes, NJ, USA) from the Flow Cytometry and Fluorescence Microscopy Unit of the UCM and Flowing Software 2 (University of Turku, Turku, Finland). For each experiment, 10^4^ cells were analyzed. Results were expressed as a percentage of the apoptotic cells over the total cells.

### 2.7. Assay for Apoptotic Pathways Identification

To identify whether samples activated apoptosis via the extrinsic or the intrinsic apoptotic pathway, DR5 and BAX expression was analyzed, respectively. First, 1 × 10^6^ HepG2 cells per well of a 6-well plate were seeded. After 24 h, cells were treated with samples 1, 5, 6, 10, 12, 14, and 16 (Table 2) at 0–100 mg/mL for 48 and 72 h. Non-treated cells were used as a negative control and cells treated with etoposide 50 µM were used as a positive control. Then, cells were detached with trypsin, counted, and two aliquots of a suspension of 1 × 10^6^ cells were separated for DR5 and BAX analysis.

For DR5 analysis, after obtaining the suspension of 1 × 10^6^ cells, cells were directly incubated with FITC Anti-DR5 antibody (DR5-01-1, ab53319) (diluted 1:15 in PBS 1X) for 45 min on ice and in darkness. Results were expressed as a percentage of the DR5-positive cells over the total cells.

For the BAX analysis, cells were fixed in 2% formaldehyde in PBS (100 µL) for 45 min at room temperature with gentle shaking. Cells were permeabilized with 0.1% Triton X-100 in 0.1% sodium citrate for 2 min on ice, washed with PBS 1X, and incubated with FITC Anti-BAX antibody (T22-A, ab139543) (diluted 1:5 in PBS 1X) for 45 min on ice and in darkness. Results were expressed as a percentage of the BAX-positive cells over the total cells at 48 and 72 h.

After incubation with the corresponding antibody, cells were washed with PBS 1X and analyzed by flow cytometry using a FACS Calibur flow cytometer (Beckton Dickinson, Franklin Lakes, NJ, USA) from the Flow Cytometry and Fluorescence Microscopy Unit of the UCM and Flowing Software 2 (University of Turku, Turku, Finland). For each experiment, 10^4^ cells were analyzed.

### 2.8. Total Extracts and Caspase Activity Assay

For the obtainment of cell lysates, HepG2 cells were seeded at 1 × 10^6^ cells per well in a 6-well plate. After 24 h, cells were treated with samples 1, 5, 6, 10, 12, 14, and 16 (Table 2) at 10–100 mg/mL depending on the sample for 48 h. Non-treated cells were used as a negative control and cells treated with etoposide 50 µM were used as a positive control. Cell lysates were prepared at 4 °C using an ice-cold total extract buffer (0.5% CHAPS, 10 mM Tris pH 7.5, 1 mM Cl_2_Mg, 1 mM EGTA, 10% glycerol, 5 mM β-mercaptoethanol, and 1 μL/mL of Protease Inhibitor Cocktail) and scraping cells off the plate. Then, cells were kept for 15 min on ice and centrifuged for another 15 min at 4 °C. After centrifugation, supernatants containing solubilized proteins were collected.

The activities of caspases 3, 8, and 9 were determined in protein extracts using the fluorogenic substrates Ac-DEVD-AMC: ALX-260-031, Ac-IETD-AMC: ALX-260-042 and Ac-LEHD-AMC: ALX-260-080, respectively, according to the manufacturer’s instructions. Briefly, 25 µL of total extract, 58 µL of total extract buffer, and 15 µL of caspase 3, 8, or 9 fluorogenic substrates were added to each well of a 96-well black plate with a clear bottom. Then, the plate was incubated at 37 °C for 1 h and the fluorescence was measured at Ex 340 nm/Em 405 nm in a fluorescence microplate reader (FLUOstar OPTIMA, BMG LABTECH, Offenburg, Germany) from the Sección Departamental de Bioquímica y Biología Molecular of the Faculty of Veterinary Medicine of the UCM. Results were expressed as the percentage of caspase activity, assuming the control was 0%.

### 2.9. Morphological Evaluation of Cell Death

HepG2 cells (1 × 10^6^ cells/mL) were exposed to etoposide (50 µM), sample 10 (CH+10PR, 25 mg/mL), sample 14 (CH+10RJ+10PR, 10 mg/mL), and sample 16 (PR, 25 mg/mL). After 48 h, cells were stained with Hoechst 33,342 (100 μg/mL) and ethidium bromide (20 μg/mL) for 5 min. Then, the dyes were washed away and the cells were examined under a fluorescence microscope (Axiostar Plus, Zeiss, Oberkochen, Germany).

### 2.10. Statistical Analyses

All means were calculated from three independent experiments and were expressed as the mean ± standard deviation (SD). One-way analysis of variance (ANOVA) was carried out and statistical comparisons of the different treatments were performed using Tukey’s test. Values of *p* < 0.05 were considered statistically significant. The Student’s *t*-test was used for statistical comparison between the different groups of treated cells and control cells. All statistical analyses were performed using the Statgraphics Centurion 19 software (Statgraphics Technologies, Inc. The Plains, VA, USA).

## 3. Results and Discussion

### 3.1. Cytotoxic Effects of Bee Product Mixtures on Human Liver Cells

The cytotoxic effects of sample 1 (TH), sample 6 (CH), sample 15 (RJ), sample 16 (PR), sample 4 (TH+2PR), sample 5 (TH+10PR), sample 10 (CH+10PR), sample 12 (TH+10RJ+10PR), and sample 14 (CH+10RJ+10PR) on liver cancer cells (HepG2) were previously published by our research group [30]. Individual samples 1 (TH), 6 (CH), 15 (RJ), and 16 (PR) at 100 mg/mL incubated for 24 h produced cytotoxic effects on HepG2 cells, significantly reducing (*p* < 0.05) the cell viability below 80%. The treatment of cells with these samples at 100 and 250 mg/mL for longer periods (48 and 72 h) caused a further reduction in cell viability.

**Figure 1:**
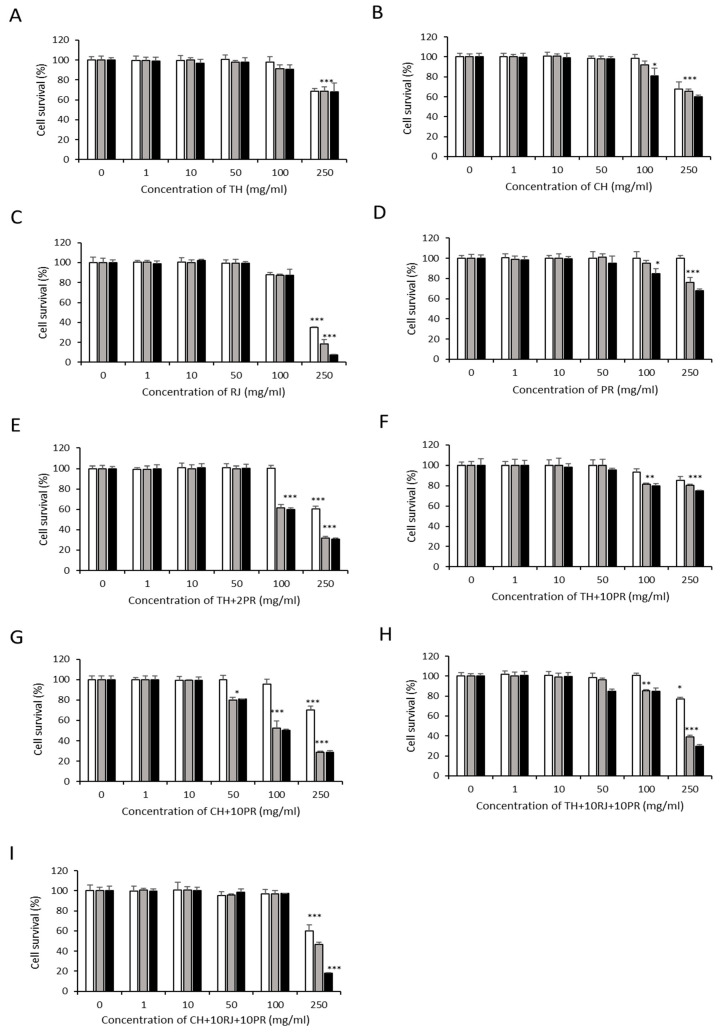
Effects of sample 1 (TH) (**A**), sample 6 (CH) (**B**), sample 15 (RJ) (**C**), sample 16 (PR) (**D**), sample 4 (TH+2PR) (**E**), sample 5 (TH+10PR) (**F**), sample 10 (CH+10PR) (**G**), sample 12 (TH+10RJ+10PR) (**H**), and sample 14 (CH+10RJ+10PR) (**I**) on WRL-68 cells’ viability by MTT assay. Cells were cultured with different doses of samples (0–250 mg/mL) for 24 (□), 48 (■), and 72 h (■). Asterisks indicate a significant difference from the control (0). * *p* ≤ 0.05, ** *p* ≤ 0.01, *** *p* ≤ 0.001.

When normal liver cells (WRL-68) (Figure 1) were treated with monofloral honey samples (samples 1, TH; and 6, CH), only the concentration of 250 mg/mL was able to reduce cell viability (>60%) (Figure 1A,B). In agreement with our results, data from a study of gelam honey on liver cells revealed that it reduced cell viability 2.8-fold more in HepG2 than in WRL-68 [35]. As for sample 1 (TH) and sample 6 (CH), artificial honey (sample 17, AH) was only cytotoxic in WRL-68 at the highest concentration (Figure S1, see Supplementary Materials). Sample 17 (AH) was found to have a weaker cytotoxic effect on HepG2 cells than the two other monofloral honeys studied at 100 mg/mL. Since it was reported that artificial honey has no effect on the survival of HepG2 cells [36,37], sugars seem not to be responsible for the cytotoxic effect observed on cancer cells. Samples 15 and 16 (RJ and PR, respectively) were more cytotoxic to HepG2 cells compared with WRL-68 cells (Figure 1C,D). Sample 15 (RJ) only reduced the survival of WRL-68 at 250 mg/mL (<40%) at all times tested, and sample 16 (PR) also decreased the survival of these cells at 250 mg/mL (76% at 48 h and 68% at 72 h).

When samples 1 (TH) and 6 (CH) were enriched with bee products, a decrease in cell viability was observed when HepG2 cells were treated with sample 4 (TH+2PR), sample 5 (TH+10PR), sample 10 (CH+10PR), sample 12 (TH+10RJ+10PR), and sample 14 (CH+10RJ+10PR) at 50 mg/mL for 72 h, and the higher concentrations of 100 and 250 mg/mL had greater cytotoxic effects [30]. In the case of WRL-68 cells, the enrichment of TH and CH with PR (samples 4, 5, and 10; Figure 1E–G) led to no effect on cell viability when cells were treated with 100 mg/mL for 24 h, but a decrease in cell viability was observed when cells were treated with this concentration for 48 and 72 h (>50%). Honey samples enriched with both bee products (samples 12, TH+10RJ+10PR; and 14, CH+10RJ+10PR) only reduced the cell viability of WRL-68 cells below 50% when the cells were treated with 250 mg/mL and for 48 and 72 h (Figure 1H,I). No previous data were published regarding the effects of RJ and PR or enriched honey mixtures on WRL-68 cells’ viability.

**Figure 2:**
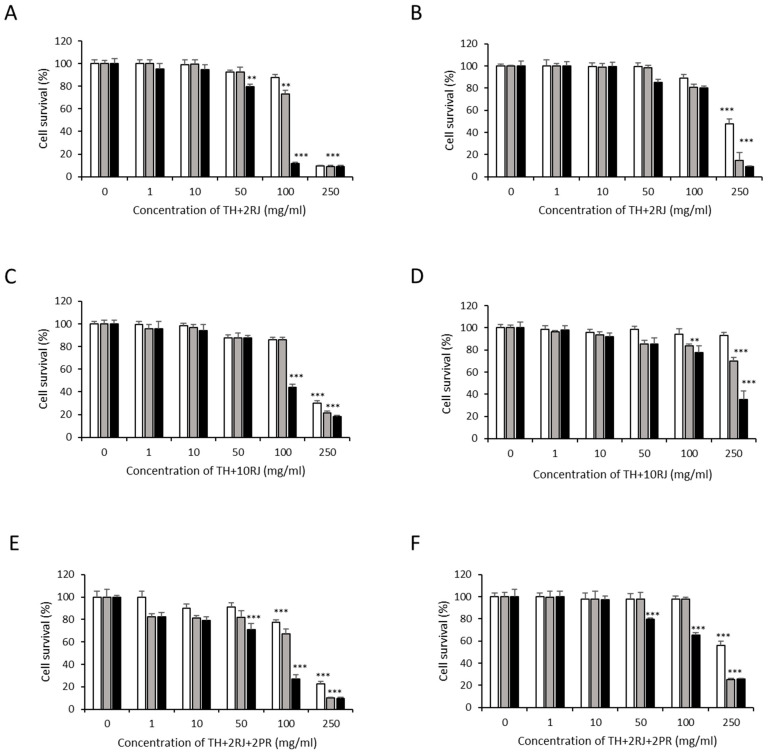
Effects on the viability of HepG2 (**A**,**C**,**E**) and WRL-68 (**B**,**D**,**F**) cells of sample 2 (TH+2RJ) (**A**,**B**), sample 3 (TH+10RJ) (**C**,**D**), and sample 11 (TH+2RJ+2PR) (**E**,**F**) by MTT assay. Cells were cultured with different doses of samples (0–250 mg/mL) for 24 (□), 48 (■), and 72 h (■). Asterisks indicate a significant difference from the control (0). ** *p* ≤ 0.01, *** *p* ≤ 0.001.

Figure 2 shows the cytotoxic effects of TH enriched with RJ (2–10%) and RJ and PR at 2% on both HepG2 and WRL-68 cells. The cytotoxic effect on HepG2 cells of sample 1 (TH) was increased with the enrichment of this sample with bee products [samples 2 (TH+2RJ), 3 (TH+10RJ), and 11 (TH+2RJ+2PR)]. For these samples, HepG2 cell viability started to decrease at 100 mg/mL and a significant cytotoxic effect (<30%) was observed when cells were treated with 250 mg/mL at all times tested (Figure 2A,C,E). When WRL-68 cells were treated with samples 2 (TH+2RJ), 3 (TH+10RJ), and 11 (TH+2RJ+2PR), no effect was observed at 100 mg/mL for 24 and 48 h, and a less aggressive cytotoxic effect was observed when used at 250 mg/mL.

**Figure 3:**
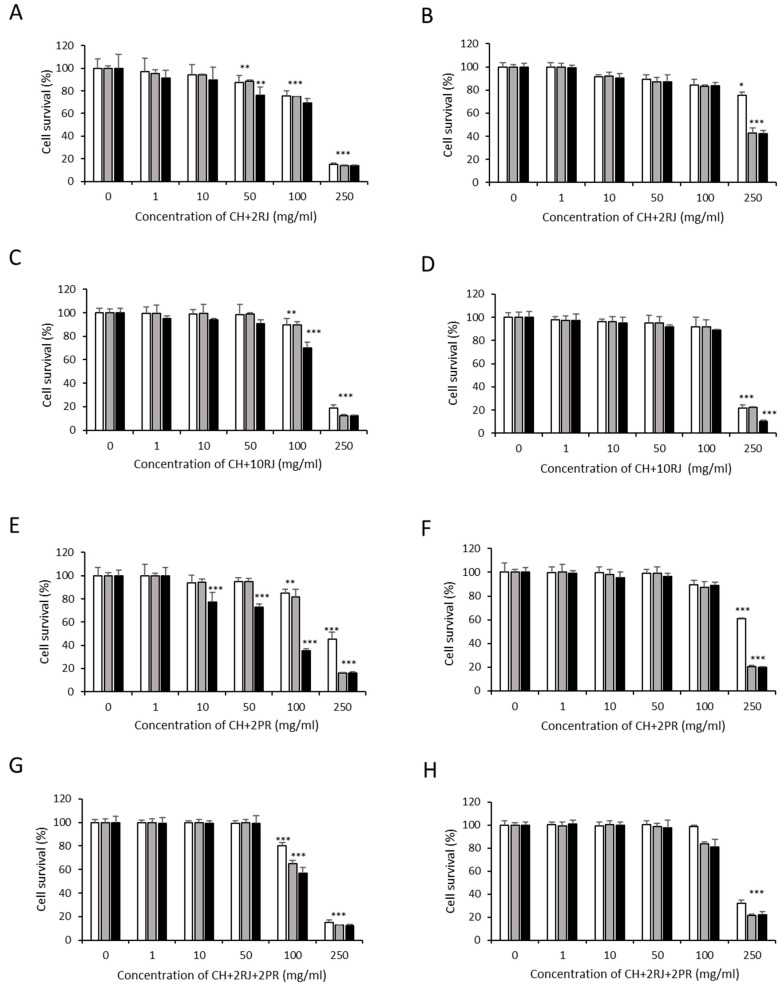
Effects on the viability of HepG2 (**A**,**C**,**E**,**G**) and WRL-68 (**B**,**D**,**F**,**H**) cells of sample 7 (CH+2RJ) (**A**,**B**), sample 8 (CH+10RJ) (**C**,**D**), sample 9 (CH+2PR) (**E**,**F**), and sample 13 (CH+2RJ+2PR) (**G**,**H**) by MTT assay. Cells were cultured with different doses of samples (0–250 mg/mL) for 24 (□), 48 (■), and 72 h (■). Asterisks indicate a significant difference from the control (0). * *p* ≤ 0.05, ** *p* ≤ 0.01, *** *p* ≤ 0.001.

Focusing on CH mixtures (Figure 3), samples 7 (CH+2RJ) and 8 (CH+10RJ) started to show a significant cytotoxic effect (*p* < 0.05) on HepG2 cells at 100 mg/mL (Figure 3A,C) that was not observed for WRL-68 cells (Figure 3B,D). These samples only produced cytotoxic effects on WRL-68 cells at 250 mg/mL and for all times tested. Sample 9 (CH+2PR) reduced HepG2 cells’ viability, even at 10 mg/mL, when incubated for 72 h (Figure 3E). This sample showed a decrease in HepG2 cell viability in a dose- and time-dependent manner. In contrast, sample 9 (CH+2PR) only reduced the cell viability of WRL-68 cells when treated at 250 mg/mL. Lastly, sample 13 (CH+2RJ+2PR) was cytotoxic for HepG2 cells at 100 and 250 mg/mL when incubated for 24, 48, and 72 h (Figure 3G), where it reduced the cell viability to 20% at the highest concentration used. In contrast, this sample only decreased the cell viability of WRL-68 cells when used at 250 mg/mL (Figure 3H).

In general, the cytotoxic effects of the antioxidant bee product mixtures were more efficient toward HepG2 cells when compared with normal liver cells (WRL-68). In line with our results, other authors described the cytotoxic effect of gelam honey and *Tinospora crispa* mixture on HepG2 and normal (WRL-68) cells, and this mixture decreased the cell viability of HepG2 cells and did not affect WRL-68 cells [38]. To the best of our knowledge, this is the first time that a comparative analysis of the cytotoxic effects of honey samples enriched with bee products was carried out in normal and cancerous hepatic cells. Further research on the effect of monofloral honey enriched with other bee products on the viability of cellular models is needed to compare the results obtained in the present study.

### 3.2. Analysis of Apoptosis Induction by Bee Product Mixtures on Human Liver Cells

**Figure 4:**
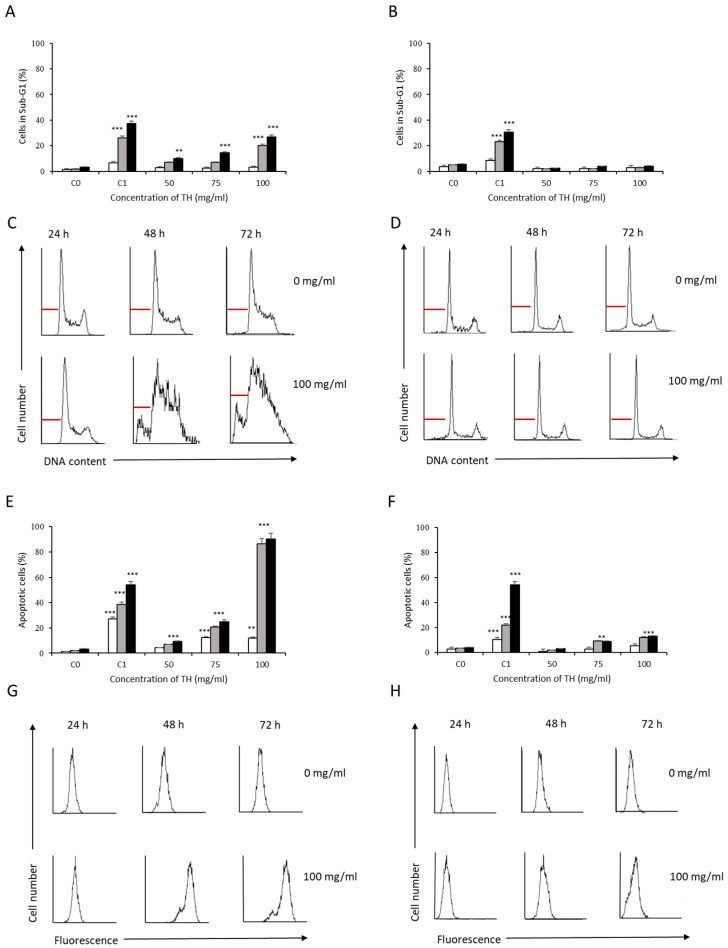
Effect of sample 1 (TH) on human liver cells apoptosis. The cell cycle was determined using flow cytometry. HepG2 (**A**) and WRL-68 (**B**) cells were treated with different doses of sample 1 (50, 75, and 100 mg/mL) for 24 (□), 48 (■), and 72 h (■). Representative histograms of the cell cycle of HepG2 (**C**) and WRL-68 (**D**) cells are shown; the red line indicates the sub-G1 phase. The percentage of apoptosis was analyzed by flow cytometry using the TUNEL assay. HepG2 (**E**) and WRL-68 (**F**) cells were cultured with different doses of sample 1 (50, 75, and 100 mg/mL) for 24 (□), 48 (■), and 72 h (■). Representative histograms of the fluorescence intensity in HepG2 (**G**) and WRL-68 (**H**) cells are presented. C_0_, untreated cells; C_1_, cells treated with etoposide (50 µM). Asterisks indicate a significant difference from the control (C_0_). ** *p* ≤ 0.01,*** *p* ≤ 0.001.

The apoptotic potential of the samples was determined via cell cycle analysis and completed using TUNEL assay. These analyses were carried out at different concentrations and for 24, 48, and 72 h of incubation, and etoposide was used as a positive control. Figure 4 shows the apoptosis induction by sample 1 (TH) on HepG2 and WRL-68 cells at 50, 75, and 100 mg/mL (24, 48, and 72 h). The cell cycle analysis exhibited an increase in HepG2 cells in the sub-G1 phase after the TH treatment (100 mg/mL, 48 and 72 h) (Figure 4A,C), but in WRL-68 cells, it did not increase the number of cells in the sub-G1 phase (Figure 4B,D). The TUNEL analysis showed that sample 1 (TH) induced apoptosis in a concentration- and time-dependent manner in HepG2 cells (Figure 4E,G), but not in normal liver cells (Figure 4F,H). The apoptotic cell population increased in HepG2 cells treated with 75 mg/mL of sample 1 (TH) for 48 (20.6%) and 72 h (25%) and was clearly evident in HepG2 cells exposed to 100 mg/mL for 48 and 72 h (86.3% and 90.2% of apoptotic cells, respectively) (Figure 4E,G). However, the percentage of apoptosis in WRL-68 cells was only 12.1% and 13%, respectively (Figure 4F,H).

**Figure 5:**
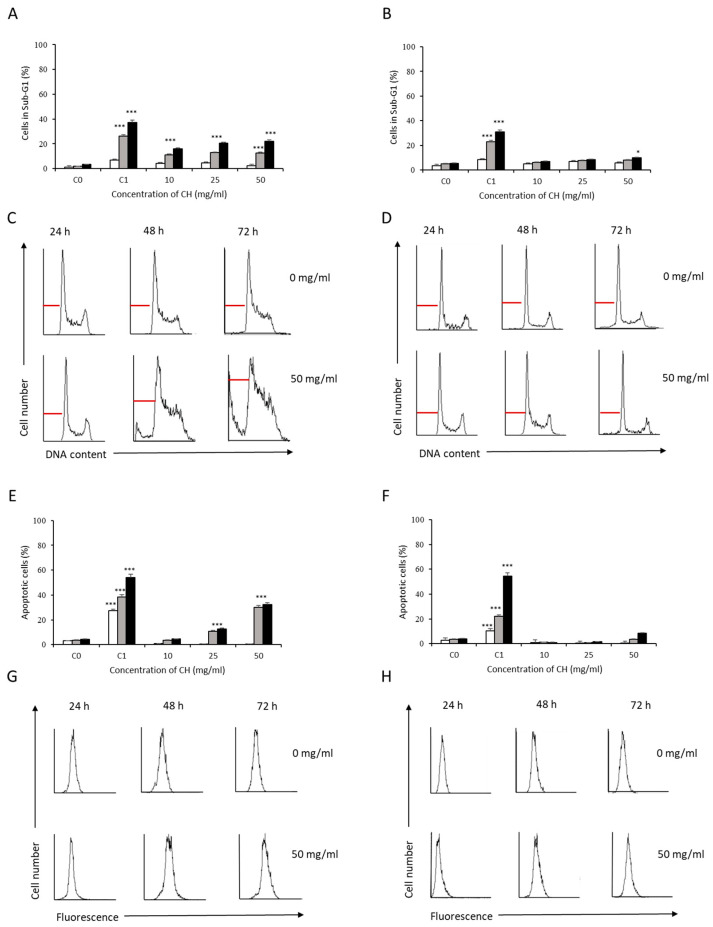
Effect of sample 6 (CH) on human liver cells apoptosis. The cell cycle was determined using flow cytometry. HepG2 (**A**) and WRL-68 (**B**) cells were treated with different doses of sample 6 (10, 25, and 50 mg/mL) for 24 (□), 48 (■), and 72 h (■). Representative histograms of the cell cycle of HepG2 (**C**) and WRL-68 (**D**) cells are shown; the red line indicates the sub-G1 phase. The percentage of apoptosis was analyzed by flow cytometry using the TUNEL assay. HepG2 (**E**) and WRL-68 (**F**) cells were cultured with different doses of sample 6 (10, 25, and 50 mg/mL) for 24 (□), 48 (■), and 72 h (■). Representative histograms of the fluorescence intensity in HepG2 (**G**) and WRL-68 (**H**) cells are presented. C_0_, untreated cells; C_1_, cells treated with etoposide (50 µM). Asterisks indicate a significant difference from the control (C_0_). * *p* ≤ 0.05,*** *p* ≤ 0.001.

As shown in Figure 5, sample 6 (CH) induced apoptosis in HepG2 cells and did not affect WRL-68 cells. In HepG2 cells, the results confirmed an increase of cells in the sub-G1 phase (Figure 5A,C). In WRL-68 cells, no changes were produced in the cell cycle (Figure 5B,D). At 50 mg/mL, one-third of the population corresponded to apoptotic HepG2 cells (30% for 48 h and 32.5% for 72 h) (Figure 5E,G), but normal liver cells were not affected (Figure 5F,H).

Other researchers also showed the apoptotic potential of honey in human hepatoma cancer cells. Al Refaey et al. demonstrated that manuka honey at a concentration of 3.46% induced apoptosis in HepG2 cells (48% of late apoptosis at 48 h) using an annexin-V/IP assay and increased the percentage of cells in the sub-G1 phase (12.1%) [39]. Jujube honey (50–80 mg/mL) also increased the percentages of total apoptotic HepG2 cells (18.6–83.3%) after treatment for 48 h [40]. The analysis of the photographs indicated that gelam honey induced apoptosis in HepG2 after 48 h of treatment with 25% of gelam honey [36]. An Alhagi honey treatment also significantly increased apoptosis of human hepatoma BEL-7402 cells (8 mg/mL for 48 h) [41]. In addition, treatment with other monofloral honey samples (rosemary, heather, tualang, acacia) also induced apoptosis in other types of cancer cells, such as leukemia, breast, or lung [42,43,44].

In contrast to sample 1 (TH) and sample 6 (CH), sample 17 (AH) did not induce apoptosis in any of the liver cell lines studied and did not increase the percentage of cells in the sub-G1 phase (Figure S2, see Supplementary Materials). These data may indicate that apoptosis of HepG2 cells is related to bioactive components of honey. In this context, several properties of honey (antioxidant, antibacterial, anti-inflammatory, or apoptotic) were attributed to phenolic compounds, especially phenolic acids and flavonoids [45,46]. In relation to apoptosis, ferulic acid, caffeic acid, benzoic acid, abscisic acid, chrysin, pinocembrin, or luteolin were reported to induce apoptosis in cancer cells (breast, bladder, cervical, lung, liver, and colorectal cancer) [47,48]. Focusing on thyme and chestnut honeys, numerous bioactive compounds were identified. Kassi et al. described that trihydroxy ketone E-4-(1,2,4-trihydroxy-2,6,6-trimethyl cyclohexyl)-but-3-en-2-one, which is a unique monoterpene in thyme honeys, reduced the viability and induced apoptosis in prostate cancer cells [49]. Another compound identified in thyme honey is rutin [50]. The flavonoid rutin inhibited cell proliferation and activated apoptosis in human glioma cells [51]. Trans-cinnamic acid, which is a compound found in chestnut honey, exerted anticancer effects against colon cancer xenografts in mice [50,52].

Comparing both monofloral honey samples, sample 6 (CH) obtained better results: at 50 mg/mL, it induced 30% (48–72 h) apoptosis, and sample 1 (TH) did not reach 10%. This difference could be related to the composition of each honey, which is related to the botanical and geographical origin [53]. Similarly, Combarros-Fuertes et al. evaluated the main bioactive compounds of Spanish thyme and chestnut honeys, among others [54]. These authors found differences in the compositions of phenolic acids and flavonoids of both honeys, which could be related to the higher apoptotic capacity of chestnut honey. The value of the total phenolic acids content was 16 times higher, and the flavonoid content was 3.6 times higher in chestnut honey [54]. Furthermore, these honeys contain quercetin, kaempferol, galangin, pinocembrin, and chrysin [54]. All of these compounds show antitumor properties. Quercetin and kaempferol induced cell cycle arrest and apoptotic cell death in liver cancer cells, and kaempferol had no effect on normal cells [55,56]. Pinocembrin suppressed proliferation and increased apoptosis in lung cancer cells in vitro, and galangin has the same effect in ovarian cancer cells [57,58]. Chrysin shows remarkable anticancer potential against several tumor cell lines, such as breast, bladder, cervix, and colon [48]. The content of quercetin, galangin, pinocembrin, and chrysin was higher in chestnut honey than in thyme honey [54].

**Figure 6:**
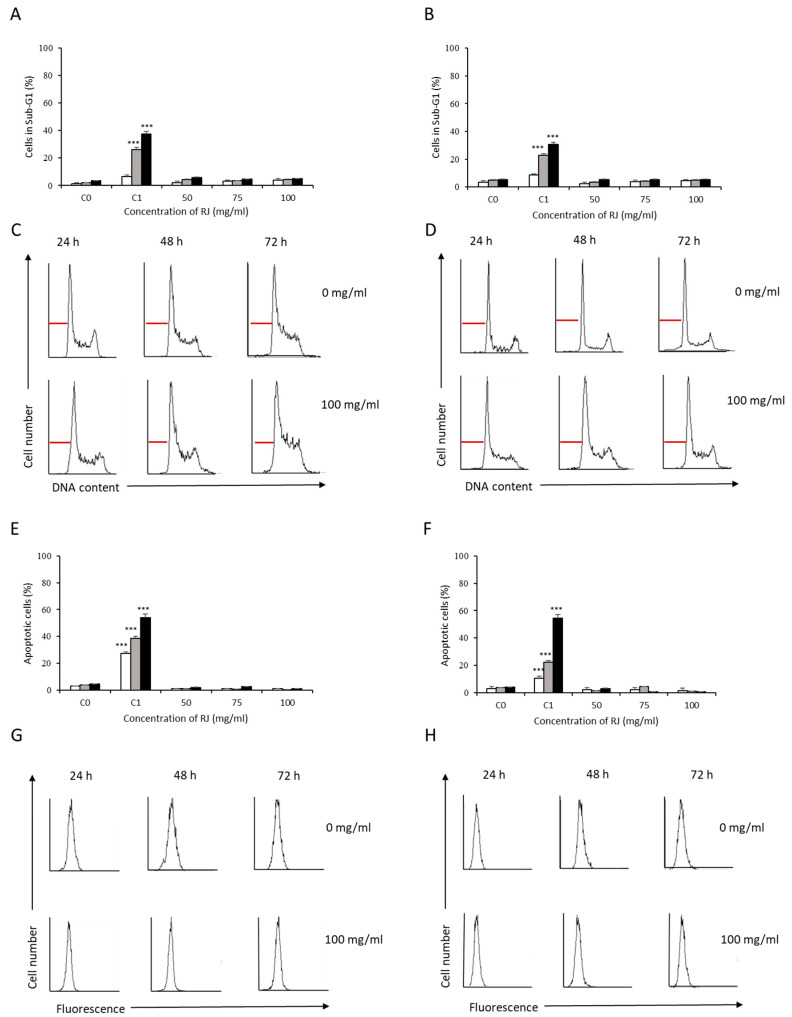
Effect of sample 15 (RJ) on human liver cell apoptosis. The cell cycle was determined using flow cytometry. HepG2 (**A**) and WRL-68 (**B**) cells were treated with different doses of sample 15 (50, 75, and 100 mg/mL) for 24 (□), 48 (■), and 72 h (■). Representative histograms of the cell cycle of HepG2 (**C**) and WRL-68 (**D**) cells are shown; the red line indicates the sub-G1 phase. The percentage of apoptosis was analyzed by flow cytometry using the TUNEL assay. HepG2 (**E**) and WRL-68 (**F**) cells were cultured with different doses of sample 15 (50, 75, and 100 mg/mL) for 24 (□), 48 (■), and 72 h (■). Representative histograms of the fluorescence intensity in HepG2 (**G**) and WRL-68 (**H**) cells are presented. C_0_, untreated cells; C_1_, cells treated with etoposide (50 µM). Asterisks indicate a significant difference from the control (C_0_). *** *p* ≤ 0.001.

The royal jelly (sample 15, RJ) (Figure 6) evaluated in this study did not increase the number of cells in the sub-G1 phase at any of the concentrations or times studied (Figure 6A–D). On the other hand, no significant increase in the percentage of apoptotic cells was observed in HepG2 and WRL-68 cells, even at 100 mg/mL, using the TUNEL assay (Figure 6E–H). In contrast, other studies reported the induction of apoptosis by royal jelly [59]. This result could be due to the different origins of the royal jelly samples and, therefore, their composition (such as lipids, proteins, and phenolic compounds) [60]. Moreover, the origin and the content of phenolic compounds in royal jelly could be affected by the season, plant species, environmental factors, and the collection time after larval transfer [60]. The apoptotic effect of royal jelly in colon and bladder cancer cells was also previously described [61,62].

**Figure 7:**
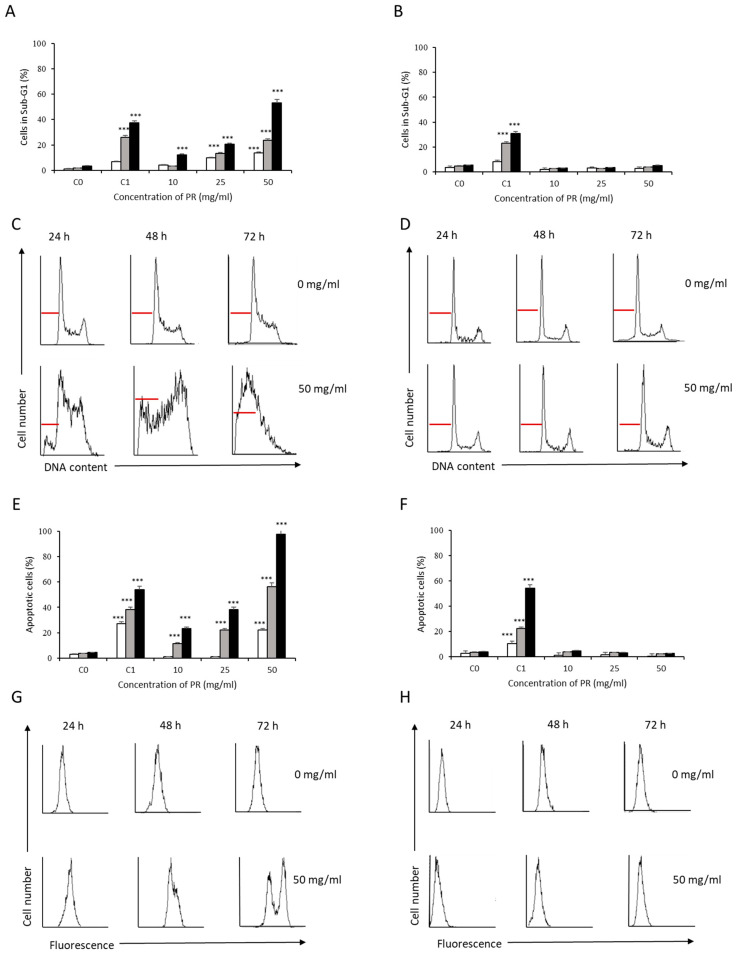
Effect of sample 16 (PR) on human liver cell apoptosis. The cell cycle was determined using flow cytometry. HepG2 (**A**) and WRL-68 (**B**) cells were treated with different doses of sample 16 (10, 25, and 50 mg/mL) for 24 (□), 48 (■), and 72 h (■). Representative histograms of the cell cycle of HepG2 (**C**) and WRL-68 (**D**) cells are shown; the red line indicates the sub-G1 phase. The percentage of apoptosis was analyzed by flow cytometry using the TUNEL assay. HepG2 (**E**) and WRL-68 (**F**) cells were cultured with different doses of sample 16 (10, 25, and 50 mg/mL) for 24 (□), 48 (■), and 72 h (■). Representative histograms of the fluorescence intensity in HepG2 (**G**) and WRL-68 (**H**) cells are presented. C_0_, untreated cells; C_1_, cells treated with etoposide (50 µM). Asterisks indicate a significant difference from the control (C_0_). *** *p* ≤ 0.001.

Figure 7 shows the effect of sample 16 (PR) on the induction of apoptosis. The cell cycle evaluation of HepG2 cells revealed an increase in the number of apoptotic cells in the sub-G1 phase after treatment with sample 16 (PR) (Figure 7A,C). After 72 h, sample 16 (PR) caused 53.05% of cells to be in the sub-G1 phase (Figure 7A). Using the TUNEL assay, a significant increase in the percentage of apoptotic HepG2 cells was observed in a time- and concentration-dependent manner (Figure 7E,G). In WRL-68 cells, sample 16 (PR) showed no apoptotic effect at any concentration or time tested and did not produce alterations in the cell cycle (Figure 7B,D,F,H). Several studies described the apoptotic effect of propolis in human hepatoma and other types of tumors. Poplar propolis from China induced apoptosis in HepG2 cells (25–100 µg/mL) at 48 h [63]. In human breast cancer cells, Turkish propolis produced cell cycle arrest in the G1 phase and elevated the amount of apoptotic cell death [64]. Zingue et al. demonstrated that Cameroonian propolis produced apoptosis in prostate cancer cells (50 µg/mL) [65]. Propolis from Al-Bahah, Saudi Arabia, increased acute T leukemia cells in the sub-G1 phase at 100 µg/mL and the number of cells in apoptosis using an annexin-V/IP assay [66]. Furthermore, 100 mg/mL of propolis induced apoptosis in colorectal cancer cells [67].

Other authors carried out LC-MS (liquid chromatography–mass spectrometry) analysis of the compounds present in propolis. The study reported the presence of ganoderic acid DM, rottlerin, (S)-beta-himachalene, and ishwarol [68]. These compounds demonstrated anticancer activity in non-small-cell lung carcinoma and breast cancer cells [68]. In addition, it was also shown that hexadecanoic acid methyl ester, which is one of the bioactive compounds of propolis, induced apoptosis in gastric cancer cells [69]. Brazilian red propolis exhibited a high cytotoxic potential on human glioblastoma, ovary, and colon cancer cell lines. Among others, the main bioactive components identified in this propolis were caffeic acid, genistein, and pinobanksin [70]. It was described that these compounds reduced proliferation and induced apoptosis in melanoma, hepatocellular carcinoma, and B-cell lymphoma cell lines, respectively [71,72,73].

The identification of components present in Spanish propolis was also reported. Extracts of propolis from Spain were mainly composed of flavonoids (pinobanksin, chrysin, pinocembrin, and galangin) and phenolic acids (caffeic acid, p-coumaric acid, and ferulic acid) [74]. Moreover, p-coumaric and ferulic acids were shown to be effective in several cancer cell lines by inducing apoptosis [75,76].

Studies on the effect of honey and bee products on apoptosis performed in normal cells are limited, and thus, it is of great interest to carry out more studies in order to be able to make a comparison.

**Figure 8:**
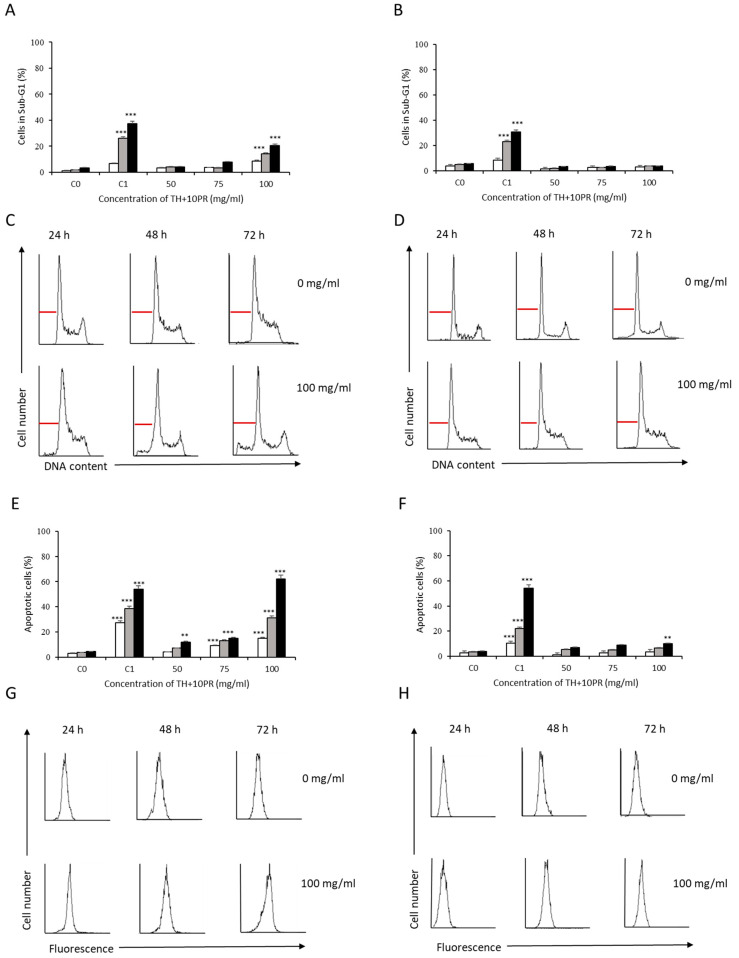
Effect of sample 5 (TH+10PR) on human liver cell apoptosis. The cell cycle was determined using flow cytometry. HepG2 (**A**) and WRL-68 (**B**) cells were treated with different doses of sample 5 (50, 75, and 100 mg/mL) for 24 (□), 48 (■), and 72 h (■). Representative histograms of the cell cycle of HepG2 (**C**) and WRL-68 (**D**) cells are shown; the red line indicates the sub-G1 phase. The percentage of apoptosis was analyzed by flow cytometry using the TUNEL assay. HepG2 (**E**) and WRL-68 (**F**) cells were cultured with different doses of sample 5 (50, 75, and 100 mg/mL) for 24 (□), 48 (■), and 72 h (■). Representative histograms of the fluorescence intensity in HepG2 (**G**) and WRL-68 (**H**) cells are presented. C_0_, untreated cells; C_1_, cells treated with etoposide (50 µM). Asterisks indicate a significant difference from the control (C_0_). ** *p* ≤ 0.01,*** *p* ≤ 0.001.

We then proceeded to evaluate the honey samples enriched with bee products (royal jelly and/or propolis, 2–10%). Sample 5 (TH+10PR) (Figure 8) increased the percentage of HepG2 cells in the sub-G1 phase (Figure 8A,C) and induced apoptosis at 100 mg/mL (15.1%–62% for 24–72 h) in these cells (Figure 8E,G). When analyzing apoptosis in normal liver cells (WRL-68), no cells in the sub-G1 were observed and only 10.2% apoptosis was detected at the highest concentration and time studied (Figure 8B,D,F,H). In this case, sample 5 (TH+10PR) did not improve the results of thyme honey alone (Figure 4A,C,E,G).

**Figure 9:**
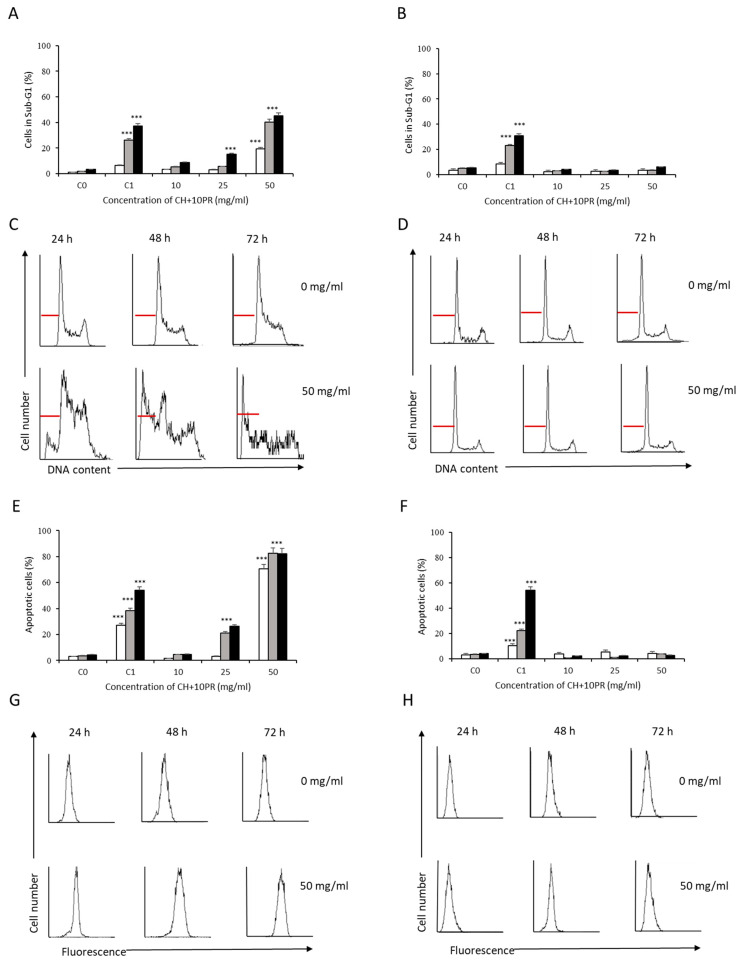
Effect of sample 10 (CH+10PR) on human liver cell apoptosis. The cell cycle was determined using flow cytometry. HepG2 (**A**) and WRL-68 (**B**) cells were treated with different doses of sample 10 (10, 25, and 50 mg/mL) for 24 (□), 48 (■), and 72 h (■). Representative histograms of the cell cycle of HepG2 (**C**) and WRL-68 (**D**) cells are shown; the red line indicates the sub-G1 phase. The percentage of apoptosis was analyzed by flow cytometry using the TUNEL assay. HepG2 (**E**) and WRL-68 (**F**) cells were cultured with different doses of sample 10 (10, 25, and 50 mg/mL) for 24 (□), 48 (■), and 72 h (■). Representative histograms of the fluorescence intensity in HepG2 (**G**) and WRL-68 (**H**) cells are presented. C_0_, untreated cells; C_1_, cells treated with etoposide (50 µM). Asterisks indicate a significant difference from the control (C_0_). *** *p* ≤ 0.001.

Figure 9 shows the apoptotic effect of sample 10 (CH+10PR). The peak of the sub-G1 phase increased at all times tested at 50 mg/mL in HepG2 cells (Figure 9A,C). The percentage of cells undergoing apoptosis increased with increasing time and concentration in cancer liver cells: at 25 mg/mL, sample 10 (CH+10PR) induced 26.3% apoptosis (72 h), and at 50 mg/mL, it increased from 70.6% (24 h) to 82.3% (72 h) (Figure 9E,G). Figure 9B,D,F,H show that normal cells were not affected at any concentration or time analyzed. Comparing the results of chestnut honey alone and the mixture with 10% propolis (sample 10, CH+10PR) in HepG2 cells, significant differences were observed. In the cell cycle, at 50 mg/mL, sample 10 (CH+10PR) increased the percentage of cells in the sub-G1 phase (Figure 9A,C) more than twice as much as chestnut honey (Figure 5A,C). At 25 mg/mL, sample 10 (CH+10PR) induced more apoptosis in tumor cells (Figure 9E,G) than chestnut honey (Figure 5E,G) at all times studied. The treatment with this mixture induced 70 times more apoptosis at 24 h and 2 times more at 48 and 72 h than chestnut honey alone at 50 mg/mL. In addition, sample 10 (CH+10PR) was much more effective than sample 5 (TH+10PR), with better results at shorter times and lower concentrations.

**Figure 10:**
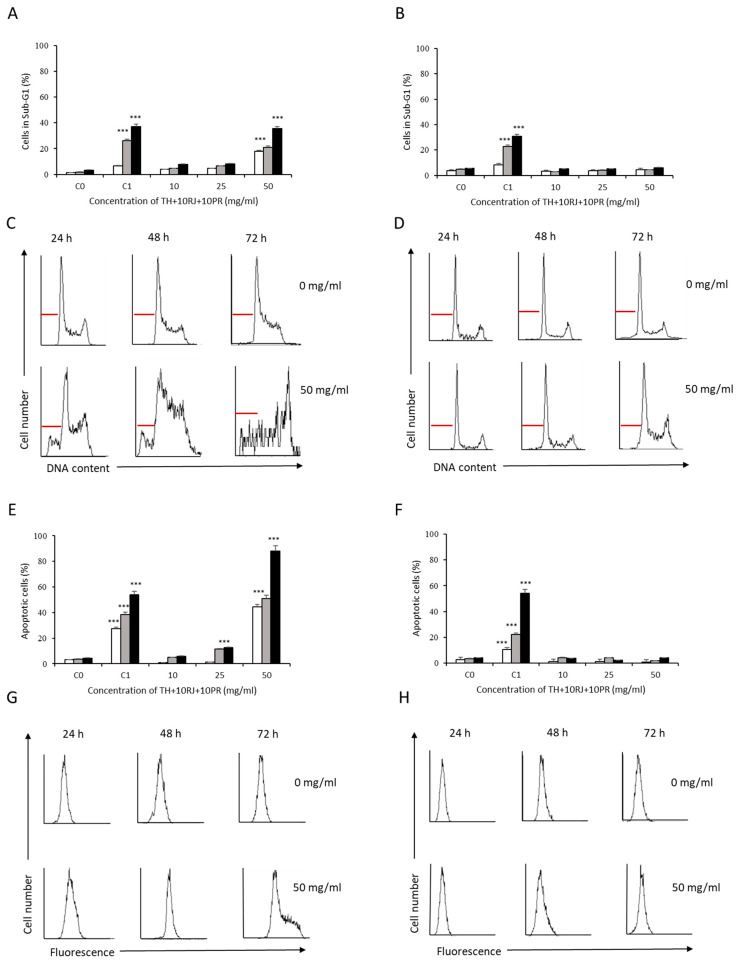
Effect of sample 12 (TH+10RJ+10PR) on human liver cell apoptosis. The cell cycle was determined using flow cytometry. HepG2 (**A**) and WRL-68 (**B**) cells were treated with different doses of sample 12 (10, 25, and 50 mg/mL) for 24 (□), 48 (■), and 72 h (■). Representative histograms of the cell cycle of HepG2 (**C**) and WRL-68 (**D**) cells are shown; the red line indicates the sub-G1 phase. The percentage of apoptosis was analyzed by flow cytometry using the TUNEL assay. HepG2 (**E**) and WRL-68 (**F**) cells were cultured with different doses of sample 12 (10, 25, and 50 mg/mL) for 24 (□), 48 (■), and 72 h (■). Representative histograms of the fluorescence intensity in HepG2 (**G**) and WRL-68 (**H**) cells are presented. C_0_, untreated cells; C_1_, cells treated with etoposide (50 µM). Asterisks indicate a significant difference from the control (C_0_). *** *p* ≤ 0.001.

The results of the apoptotic effect of sample 12 (TH+10RJ+10PR) are shown in Figure 10. Using 50 mg/mL significantly increased the sub-G1 peak at 24, 48, and 72 h in HepG2 cells (Figure 10A,C). The apoptotic effect of this sample in HepG2 cells was observed at a concentration of 50 mg/mL at 24, 48, and 72 h (44.3%, 51%, and 88%, respectively) (Figure 10E,G). Normal liver cells were not affected by sample 12 (TH+10RJ+10PR) (Figure 10B,D,F,H). This mixture improved the result of thyme honey alone (Figure 4A,C,E,G), significantly increasing apoptosis at lower concentrations (50 mg/mL for sample 12 (TH+10RJ+10PR) vs. 100 mg/mL for thyme honey) in cancer liver cells.

**Figure 11:**
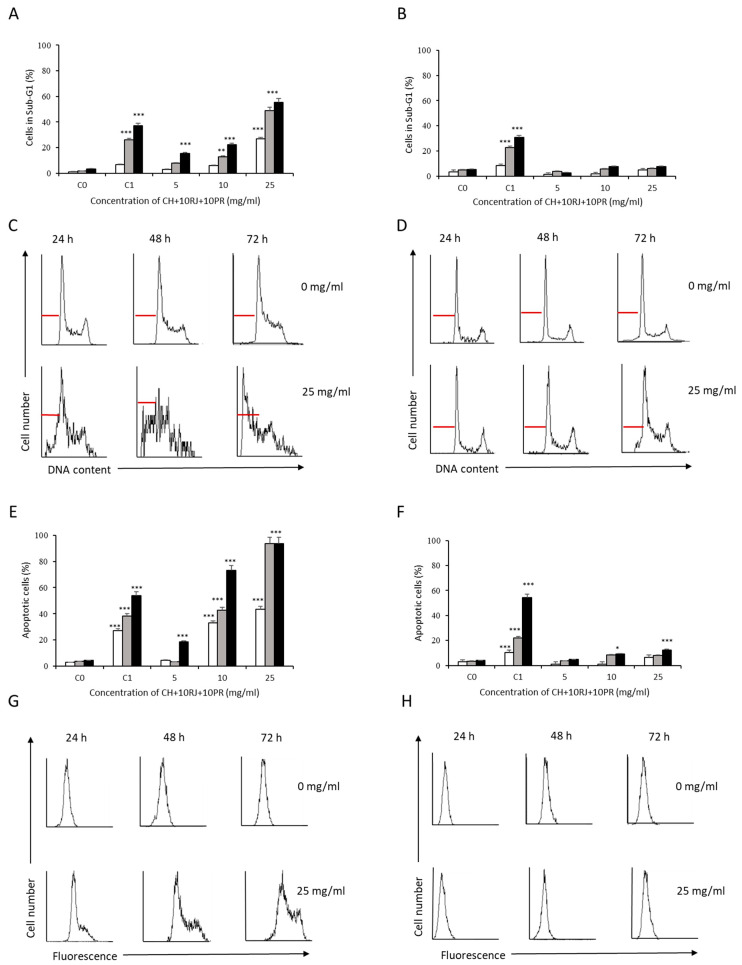
Effect of sample 14 (CH+10RJ+10PR) on human liver cell apoptosis. The cell cycle was determined using flow cytometry. HepG2 (**A**) and WRL-68 (**B**) cells were treated with different doses of sample 14 (5, 10, and 25 mg/mL) for 24 (□), 48 (■), and 72 h (■). Representative histograms of the cell cycle of HepG2 (**C**) and WRL-68 (**D**) cells are shown; the red line indicates the sub-G1 phase. The percentage of apoptosis was analyzed by flow cytometry using the TUNEL assay. HepG2 (**E**) and WRL-68 (**F**) cells were cultured with different doses of sample 14 (5, 10, and 25 mg/mL) for 24 (□), 48 (■), and 72 h (■). Representative histograms of the fluorescence intensity in HepG2 (**G**) and WRL-68 (**H**) cells are presented. C_0_, untreated cells; C_1_, cells treated with etoposide (50 µM). Asterisks indicate a significant difference from the control (C_0_). * *p* ≤ 0.05,** *p* ≤ 0.01,*** *p* ≤ 0.001.

The apoptosis-inducing capacity of sample 14 (CH+10RJ+10PR) (Figure 11) was also analyzed. After treatment with sample 14 (CH+10RJ+10PR) (10 and 25 mg/mL), an increase in the population of cells in the sub-G1 phase of the cell cycle was observed in HepG2 cells (Figure 11A,C). Figure 11E,G show that sample 14 (CH+10RJ+10PR) increased the percentage of apoptotic cancer cells at 5 mg/mL after 72 h (18.5%) at 10 mg/mL and 25 mg/mL after 24–72 h (33–73.2% and 43.6–93.6%, respectively). In addition, treatment of WRL-68 cells with sample 14 (CH+10RJ+10PR) did not significantly affect apoptosis (Figure 11B,D,H). Clearly, this mixture improved the results of chestnut honey alone (Figure 5A,C,E,G) regarding apoptosis (at lower concentrations: 10 and 25 mg/mL of sample 14 (CH+10RJ+10PR) vs. 50 mg/mL of chestnut honey) in cancer liver cells.

Of all the studied mixtures, only those enriched with 10% propolis showed apoptotic effects in HepG2 cells and had no effect on normal cells, namely, sample 5 (TH+10PR), sample 10 (CH+10PR), sample 12 (TH+10RJ+10PR), and sample 14 (CH+10RJ+10PR). Sample 2 (TH+2RJ), sample 3 (TH+10RJ), sample 4 (TH+2PR), sample 11 (TH+2RJ+2PR), sample 7 (CH+2RJ), sample 8 (CH+10RJ), sample 9 (CH+2PR), and sample 13 (CH+2RJ+2PR) did not induce apoptosis at the concentration of 100 mg/mL at 72 h on human cancer and normal liver cells (4.13%–8.53% and 3.24%–8.91%, respectively) (Table S1, see Supplementary Materials).

**Figure 12:**
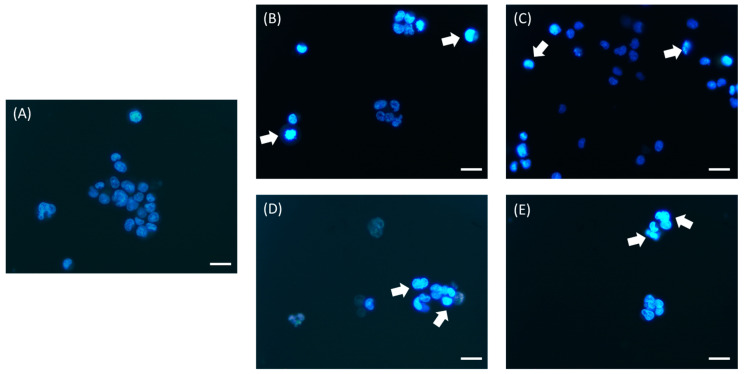
Representative images (40×) of morphological changes of nuclear chromatin in HepG2 cells stained with Hoechst 33,342 and ethidium bromide. Cells were plated in the absence (**A**) or the presence of etoposide (50 µM) (**B**), sample 10 (CH+10PR, 25 mg/mL) (**C**), sample 14 (CH+10RJ+10PR, 10 mg/mL) (**D**), and sample 16 (PR, 25 mg/mL) (**E**) for 48 h. White arrows indicate apoptotic cells. Scale bar = 50 µm.

Cell morphology was also analyzed under a fluorescence microscope using the samples that induced a higher percentage of apoptosis in tumor cells but not in normal cells. We observed significant nuclear chromatin condensation and fragmentation in liver cancer cells treated with chestnut honey mixtures and propolis (Figure 12).

To the best of our knowledge, this is the first study to describe the differential apoptotic properties of honey mixtures with royal jelly and propolis in liver human cancer and normal cells. Other studies described the apoptotic potential of mixtures of honey with other natural products in cancer cells. In HepG2 cells, gelam honey and *Tinospora crispa* mixture also induced apoptosis [38]. Hassan et al. demonstrated that bee honey + *Nigella sativa* mixture was effective at apoptosis induction in human hepatoma cells [36].

In general, propolis showed a high apoptotic potential compared with the thyme and chestnut honeys and royal jelly evaluated. Moreover, the mixtures with 10% propolis showed higher apoptotic activity than the thyme and chestnut honeys alone, except for sample 5 (TH+10PR). These mixtures were the ones that showed the highest antioxidant capacity and protective effect against benzo(a)pyrene-induced DNA damage, in our previous works, together with propolis [29,30]. In agreement, astaxanthin, which is a strong antioxidant carotenoid, showed an apoptotic effect in colorectal cancer cells [77]. Ellagic acid also exhibited antioxidant activity, as well as apoptotic potential in osteogenic sarcoma cells [78]. An aqueous extract of *Urtica dioica* showed antioxidant and apoptotic effects in breast cancer cells [79].

In addition, apoptosis values of chestnut honey mixtures were higher than those of thyme honey. Of all the samples, chestnut honey enriched with 10% royal jelly and 10% propolis (sample 14, CH+10RJ+10PR) showed the highest apoptotic effect. This mixture induced apoptosis in HepG2 tumor cells at the lowest concentrations without affecting normal cells.

### 3.3. Evaluation of the Apoptotic Mechanisms of Selected Samples on HepG2 Cells

Samples that induced apoptosis in liver tumor cells and not in normal cells were selected for the study of their apoptotic mechanisms [samples 1 (TH), 5 (TH+10PR), 6 (CH), 10 (CH+10PR), 12 (TH+10RJ+10PR), 14 (CH+10RJ+10PR), and 16 (PR)]. Apoptosis can be activated by two different pathways, namely, the extrinsic and intrinsic (death receptor and mitochondrial) pathways [80]. Based on previous data, we studied whether the samples selected activated TRAILR2 (Tumor-necrosis-factor-Related Apoptosis-Inducing Ligand Receptor 2) or DR5 in HepG2 cells. This membrane receptor is normally activated in liver tumor cells that enter apoptosis and in which the extrinsic pathway is involved [81,82,83].

As shown in Table 3, treatment with all samples increased the number of DR5-positive cells. Sample 14 (CH+10RJ+10PR) and sample 16 (PR) showed similar values at 48 and 72 h, and in the other samples, the values were higher at 72 h. Sample 6 (CH) at half the concentration of sample 1 (TH) showed higher values. As mentioned above, this difference could have been due to the different compositions of thyme and chestnut honeys, such as phenolic acids and flavonoids. Chrysin is the most common flavone in monofloral honeys and several studies showed that it upregulates DR5 expression in cervical cancer cells and HepG2 cells, inducing apoptosis [84,85,86]. Similarly, Combarros-Fuertes et al. found differences in chrysin concentration between Spanish thyme and chestnut honeys, with values of 0.04 and 0.34, respectively [54]. Differences were also found in the content of pinocembrin and cis,trans-abscisic acid, and values were higher in chestnut honey [54]. On the other hand, sample 16 (PR) exhibited nearly 100% DR5-positive cells at 48 and 72 h. In addition to honey, chrysin is also found in propolis [48]. A study reported that major components identified in Spanish propolis were caffeic acid, ferulic acid, chrysin, and pinocembrin, among others, with chrysin being the most representative [87].

Sample 5 (TH+10PR) showed a higher number of DR5-positive cells than thyme honey alone. The percentage of DR5-positive cells with sample 12 (TH+10RJ+10PR) was slightly lower than thyme honey alone, but thyme honey was used at twice the concentration. Chestnut honey mixtures with 10% propolis showed better results than chestnut honey, significantly increasing the percentage of DR5-positive cells. At 48 h, sample 10 (CH+10PR) showed a higher percentage of DR5-positive cells than chestnut honey alone, particularly at 72 h. Sample 14 (CH+10RJ+10PR) significantly increased the value of chestnut honey alone at 48 and 72 h. Chestnut honey mixtures showed higher percentages of DR5-positive cells than thyme honey mixtures and at lower concentrations.

One of the most important molecules involved in the intrinsic pathway is BAX. BAX, which is a pro-apoptotic member of the Bcl-2 family, is implicated in mitochondrial outer membrane permeabilization, which is a point of no return in apoptosis [88]. Table 3 showed that sample 1 (TH) and sample 6 (CH) did not show BAX-positive cells at 48 h; however, the percentage increased at 72 h (TH: 20.21% and CH: 17.60%). Other authors described that manuka honey activated BAX in HepG2 cells, as well as chrysin in human lung adenocarcinoma cells [39,89]. Sample 16 (PR) was the sample that revealed the highest percentage of BAX-positive cells (89.78% and 99.99% at 48 and 72 h, respectively). Motomura et al. described that propolis induced apoptosis in human leukemic cells without regulating BAX levels [90], although other authors demonstrated the implication of propolis treatment on BAX levels. Brazilian, Kermanian, and Turkish propolis produced an overexpression of the gene/protein BAX in human lung, acute myeloblastic leukemia, and breast cancer cells [64,91,92].

Focusing on the mixtures, sample 5 (TH+10PR) showed an increase in the percentage of BAX-positive cells at 72 h, with a value equivalent to thyme honey. The value of BAX-positive cells with sample 12 (TH+10RJ+10PR) was slightly lower than thyme honey alone, but thyme honey (sample 1) was used at 100 mg/mL and sample 12 (TH+10RJ+10PR) at 50 mg/mL. Sample 10 (CH+10PR) showed 99.62% BAX-positive cells at 72 h and chestnut honey alone showed a value of 17.60%. Sample 14 (CH+10RJ+10PR) increased the percentage of BAX-positive cells at 48 h (32.33%) and 72 h (72.73%). Sample 14 (CH+10RJ+10PR) also improved the results of chestnut honey alone. As for DR5, chestnut honey mixtures showed higher percentages of BAX-positive cells than thyme honey mixtures, even at lower concentrations.

Etoposide increased the percentage of DR5- and BAX-positive cells at 48 h and 72 h (Table 3). At 48 h, the value of DR5-positive cells was higher than BAX, but at 72 h, the values were similar. At 72 h, sample 5 (TH+10PR), sample 10 (CH+10PR), sample 14 (CH+10RJ+10PR), and sample 16 (PR) showed higher values than etoposide in DR5-positive cells.

According to these results, both apoptotic pathways are involved in the induction of apoptosis after treatment with the samples in liver cancer cells. Thyme honey mixtures (sample 5, TH+10PR, and sample 12, TH+10RJ+10PR) equaled or increased the results of thyme honey alone, and chestnut honey mixtures (sample 10, CH+10PR, and sample 14, CH+10RJ+10PR) increased those of chestnut honey alone in all tests.

Caspases are effector endoproteases that play a key role in apoptosis. These molecules can be classified according to their mechanism of action into initiators (such as caspases 8 and 9) and executioners (such as caspase 3) [93]. We examined the activation of caspases 8, 9, and 3 after treatment with samples that showed an apoptotic effect in HepG2 cells (Table 4) using caspase-specific substrates. As shown in Table 4, caspase 8 activity was significantly induced by all the samples selected (83.73%–93.68%). Interestingly, treatment with the studied samples also induced activation of caspase 9 (15.44%–19.71%). The data obtained for caspase 3 revealed the activation of this executioner caspase by the samples (44.06%–50.28%). At the concentration and time analyzed, the caspase that showed greater activation was caspase 8.

The initiator caspase 8 is responsible for the extrinsic pathway, and caspase 9 for the intrinsic pathway. Both caspases 8 and 9 activate other executioner caspases, such as caspase 3 [93]. Considering these results, it could be confirmed that caspases of both apoptotic pathways were involved after treatment with the samples.

After analyzing the induction of the death receptor DR5 and BAX, as well as the activation of caspases 8, 9, and 3, our results suggested that apoptosis occurs via the extrinsic pathway through death signals received from outside the cell, and the intrinsic pathway occurs due to intracellular signals.

**Figure 13:**
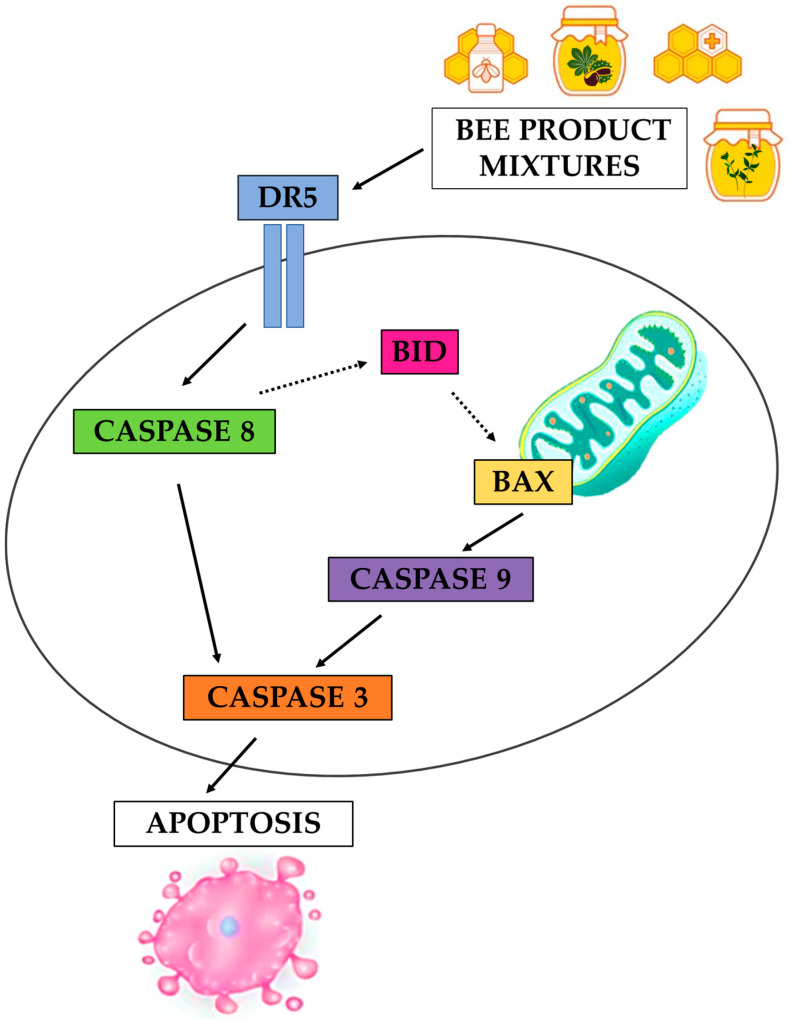
Proposed mechanism for the effect of bee product mixtures on the induction of apoptosis in liver cancer cells.

The proposed mechanism (Figure 13) for the apoptosis-inducing samples [samples 1 (TH), 5 (TH+10PR), 6 (CH), 10 (CH+10PR), 12 (TH+10RJ+10PR), 14 (CH+10RJ+10PR), and 16 (PR)] is that treatment with these samples first activates the death receptor DR5 (other receptors, such as DR4 or Fas, may also be activated), which is involved in the activation of caspase 8 [80]. Bid is a pro-apoptotic Bcl-2 family member and can be activated by caspase 8, which provides a connection between the extrinsic and intrinsic pathways [33]. The active caspase 8 after treatment with the samples could activate Bid and this activated the intrinsic pathway, which is why we observed the increase in the percentage of BAX-positive cells. BAX causes the outer mitochondrial membrane to become permeable and cytochrome c is released into the cytosol and can activate caspase 9, together with Apaf-1 (apoptotic peptidase activating factor-1) [80]. Finally, both caspases 8 and 9 activate executioner caspase 3 [80]. We propose this mechanism based on the results: the values of DR5-positive cells were higher than BAX-positive cells, the activity of caspase 8 was greater than caspase 9, and caspase 3 values were intermediate between caspases 8 and 9.

In addition, it was described that chrysin activates death receptors and Bid, inducing apoptosis [48]. Other researchers reported that pinocembrin activated the extrinsic pathway via death receptors and caffeic acid activated caspase 8 and BAX [94,95]. BAX-dependent apoptosis during liver cell damage is important for cancer prevention [96]. In addition, evasion of apoptosis is commonly observed during liver cancer development and BAX is downregulated [97]. BAX activation is key to inducing apoptosis in liver tumors and all the samples analyzed increased BAX in HepG2 cells.

In general, mixtures showed better results than honey samples alone regarding apoptosis, and CH (sample 6) and its mixtures were also more effective compared with TH (sample 1) and its mixtures. This could be explained by the difference in the composition of the two kinds of honey: CH has higher chrysin and pinocembrin contents than TH [54]. The difference in composition, especially in phenolic compounds, is related to the other biological activities of honey [97]. This composition could also be related to the apoptotic activity of propolis. The mixtures that showed induction of apoptosis were those that showed the highest total phenolic content and antioxidant capacity in our previous study: mixtures of TH and CH enriched with 10% propolis [samples 5 (TH+10PR), 10 (CH+10PR), 12 (TH+10RJ+10PR), and 14 (CH+10RJ+10PR)], and propolis (sample 16) [29].

Several types of honey induce apoptosis via ROS (reactive oxygen species)-dependent, although others do so independently [42,98]. These mixtures and propolis induced apoptosis via ROS-independent since the samples reduced ROS levels over time in HepG2 cells [29]. Thyme and chestnut honeys, propolis, and mixtures of thyme and chestnut honeys enriched with 10% propolis also presented antioxidant capacity and protective activity against DNA damage [29,30].

Moreover, the samples used in this study reduced cell viability more effectively in HepG2 cells than in normal cells and produced apoptosis in cancer cells while not affecting normal cells, which is critical in approaching tumor therapy since most chemotherapeutic agents induce apoptosis not only in tumor cells but also in normal cells. In HepG2 cells, treatment with manuka honey and Doxorubicin had a synergistic effect on the induction of apoptosis [39]. In a different study, Algerian propolis potentiated the apoptotic effect of Doxorubicin in human pancreatic cancer cells [99].

Overall, the antioxidant bee product mixtures with apoptotic properties could be used as an adjuvant or dietary supplement as complementary medicine in combination with chemotherapy treatments to increase the anticancer effects of the conventional treatment and to reduce its cytotoxic side effects. Further clinical studies are needed to study the efficacy of these antioxidant bee product mixtures as complementary medicine. In addition, these samples could be used as natural sweeteners enriched in antioxidants with great added value and also to reduce the use of additives in processed foods [29].

Besides the use of these mixtures as complementary medicine for cancer treatment, the enrichment of monofloral honey with bee products is a promising way to support local beekeepers, help preserve the bees, and therefore, contribute to achieving the Sustainable Development Goals (goals 1, 2, 8, and 15) proposed by the United Nations.

## 4. Conclusions

The present work evaluated for the first time the cytotoxic and apoptotic effects of thyme and chestnut honeys and their mixtures with royal jelly and/or propolis (2–10%) in human liver cancer and normal cells. The studied samples had no or very little apoptotic effect on normal cells. Antioxidant honey mixtures enriched with bee products enhanced the apoptotic capacity of honey alone. Apoptosis involved the extrinsic and intrinsic pathways, with activation of DR5; BAX; and caspases 8, 9, and 3. Of all the samples, chestnut honey enriched with 10% royal jelly and 10% propolis (sample 14, CH+10RJ+10PR) showed the highest apoptotic effect. The enrichment of monofloral honey with bee products could be used together with conventional anticancer treatments as a dietary supplement without side effects. On the other hand, it could be included in the diet as a natural sweetener with high added value.

## Figures and Tables

**Table 1:** Honey and bee product samples.

Samples	Scientific and Common Names	Type	Family	Geographic Region
Chestnut honey	*Castanea sativa* Chestnut	Monofloral (47.19%)	Fagaceae	Spain, Toledo (4°44′48″ W, 40°06′26″ N)
Thyme honey	*Thymus* spp. Thyme	Monofloral (64.98%)	Lamiaceae	Spain, Zamora (5°44′40″ W, 41°30′22″ N)
Royal jelly	-	-	-	France
Propolis tincture *	-	-	-	Spain, Zamora (5°44′40″ W, 41°30′22″ N)

* Propolis extract dissolved in 70% organic ethanol.

**Table 2:** Honey and bee products samples and mixtures.

Sample No.	Description	Code
1	Thyme honey	TH
2	Thyme honey + 2% royal jelly	TH+2RJ
3	Thyme honey + 10% royal jelly	TH+10RJ
4	Thyme honey + 2% propolis	TH+2PR
5	Thyme honey + 10% propolis	TH+10PR
6	Chestnut honey	CH
7	Chestnut honey + 2% royal jelly	CH+2RJ
8	Chestnut honey + 10% royal jelly	CH+10RJ
9	Chestnut honey + 2% propolis	CH+2PR
10	Chestnut honey + 10% propolis	CH+10PR
11	Thyme honey + 2% royal jelly + 2% propolis	TH+2RJ+2PR
12	Thyme honey + 10% royal jelly + 10% propolis	TH+10RJ+10PR
13	Chestnut honey + 2% royal jelly + 2% propolis	CH+2RJ+2PR
14	Chestnut honey + 10% royal jelly + 10% propolis	CH+10RJ+10PR
15	Royal jelly	RJ
16	Propolis	PR
17	Artificial honey	AH

**Table 3:** Effect of selected samples (1, 5, 6, 10, 12, 14, and 16) and etoposide on DR5 and BAX activation in HepG2 cells, as evaluated using flow cytometry at 48 and 72 h.

Sample	Code	Concentration (mg/mL)	DR5-Positive Cells 48 h (%)	DR5-Positive Cells 72 h (%)	BAX-Positive Cells 48 h (%)	BAX-Positive Cells 72 h (%)
Control	-	-	4.19 ± 0.68	4.87 ± 0.73	4.86 ± 0.97	5.79 ± 0.52
1	TH	100	16.19 ± 0.21 ***	25.97 ± 2.09 ***	7.53 ± 1.17	20.21 ± 1.49 ***
5	TH+10PR	100	25.83 ± 3.02 ***	68.28 ± 2.78 ***	6.01 ± 1.11	19.46 ± 2.26 ***
6	CH	50	27.53 ± 2.60	28.58 ± 3.19 ***	3.45 ± 0.98	17.60 ± 2.03 ***
10	CH+10PR	25	33.10 ± 2.39 ***	98.08 ± 1.54 ***	3.01 ± 1.84	99.62 ± 0.16 ***
12	TH+10RJ+10PR	50	10.93 ± 1.07 ***	19.18 ± 2.51 ***	2.12 ± 0.43	15.83 ± 1.20 ***
14	CH+10RJ+10PR	10	84.82 ± 0.68 ***	89.99 ± 1.32 ***	32.33 ± 2.12 ***	72.73 ± 2.05 ***
16	PR	25	99.97 ± 0.07 ***	99.77 ± 0.14 ***	89.78 ± 2.60 ***	99.99 ± 0.06 ***
Etoposide	-	50 µM	30.26 ± 1.34 ***	47.78 ± 2.58 ***	20.62 ± 2.24 ***	46.95 ± 1.14 ***

Data are expressed as the means ± standard deviation (n = 3). Asterisks indicate significant differences from the untreated cells (control). *** *p* ≤ 0.001.

**Table 4:** Activation of caspases 8, 9, and 3 by the selected samples (1, 5, 6, 10, 12, 14, and 16) and etoposide at 48 h.

Sample	Code	Concentration (mg/mL)	Caspase 8 (%)	Caspase 9 (%)	Caspase 3 (%)
1	TH	100	93.48 ± 4.56 ***	17.42 ± 2.77 ***	45.34 ± 2.99 ***
5	TH+10PR	100	89.75 ± 4.15 ***	15.87 ± 2.54 ***	44.28 ± 3.78 ***
6	CH	50	93.68 ± 2.78 ***	16.35 ± 2.65 ***	47.13 ± 3.11 ***
10	CH+10PR	25	83.75 ± 2.47 ***	15.44 ± 2.04 ***	45.07 ± 2.73 ***
12	TH+10RJ+10PR	50	88.40 ± 3.37 ***	15.92 ± 2.40 ***	50.28 ± 3.27 ***
14	CH+10RJ+10PR	10	86.12 ± 3.54 ***	19.71 ± 2.39 ***	48.96 ± 2.76 ***
16	PR	25	83.73 ± 3.38 ***	15.90 ± 2.38 ***	44.06 ± 3.19 ***
Etoposide	-	50 µM	92.98 ± 3.76 ***	22.36 ± 2.26 ***	70.78 ± 3.41 ***

Data are expressed as the means ± standard deviation (n = 3). The results are expressed as the percentage of caspase activity, assuming the control was 0%. Asterisks indicate significant differences from the untreated cells. *** *p* ≤ 0.001.

## Data Availability

The data is contained within the article and Supplementary Materials.

## Supplementary Materials

The following supporting information can be downloaded from https://www.mdpi.com/article/10.3390/antiox12030615/s1. Figure S1: Effect of sample 17 (AH) on HepG2 (A) and WRL-68 (B) cells viability by MTT assay. Figure S2: Effect of sample 17 (AH) on human liver cells apoptosis. Table S1: Effect of samples 2, 3, 4, 7, 8, 9, 11, and 13 (100 mg/mL at 72 h) on human liver cells apoptosis analyzed by flow cytometry using the TUNEL assay.
