# Supplementary material for: Differential Apoptotic Effects of Bee Product Mixtures on Normal and Cancer Hepatic Cells
Source: Antioxidants (Basel). 2023 Mar 2;12(3):615. doi: 10.3390/antiox12030615 (PMC10045410; doi:10.3390/antiox12030615)
Supplement: Supplementary file 1 [file antioxidants-12-00615-s001.zip › antioxidants-2219787-supplementary.pdf]

Differential apoptotic effects of bee product mixtures on normal and cancer hepatic cells

Vanesa Sánchez-Martín[1], Paloma Morales[1], Amaia Iriondo-DeHond[1], Xavier F. Hospital[2], Manuela Fernández[2], Eva Hierro[2] and Ana I. Haza[1*]

1 Departamento de Nutrición y Ciencia de los Alimentos, Sección Departamental de Nutrición y Ciencia de los Alimentos, Facultad de Veterinaria, Universidad Complutense, 28040 Madrid, Spain
2 Departamento de Farmacia Galénica y Tecnología de los Alimentos, Sección Departamental de Farmacia Galénica y Tecnología de los Alimentos, Facultad de Veterinaria, Universidad Complutense, 28040 Madrid, Spain
* Correspondence: hanais@ucm.es; Tel.: +34-91-394-3749

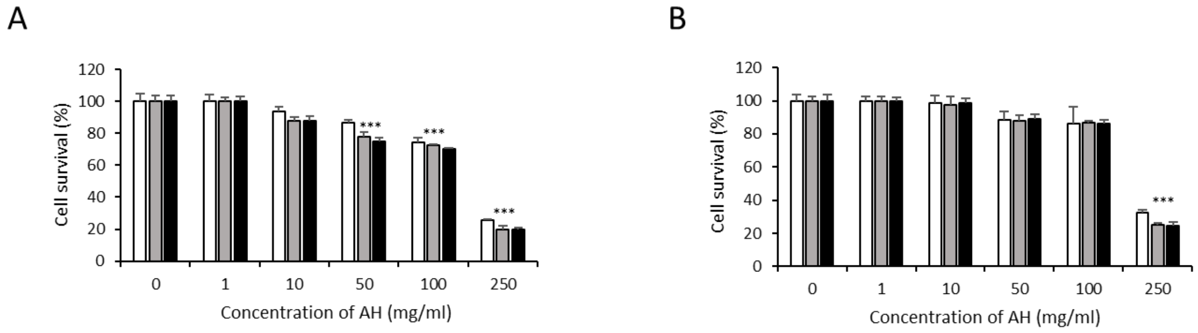

Figure S1. Effect of sample 17 (AH) on HepG2 (A) and WRL-68 (B) cells viability by MTT assay. Cells were cultured with different doses of samples (0-250 mg/ml) for 24 (□), 48 (■), and 72 hours (■). Asterisks indicate a significant difference from the control (0). ***P≤0.001.

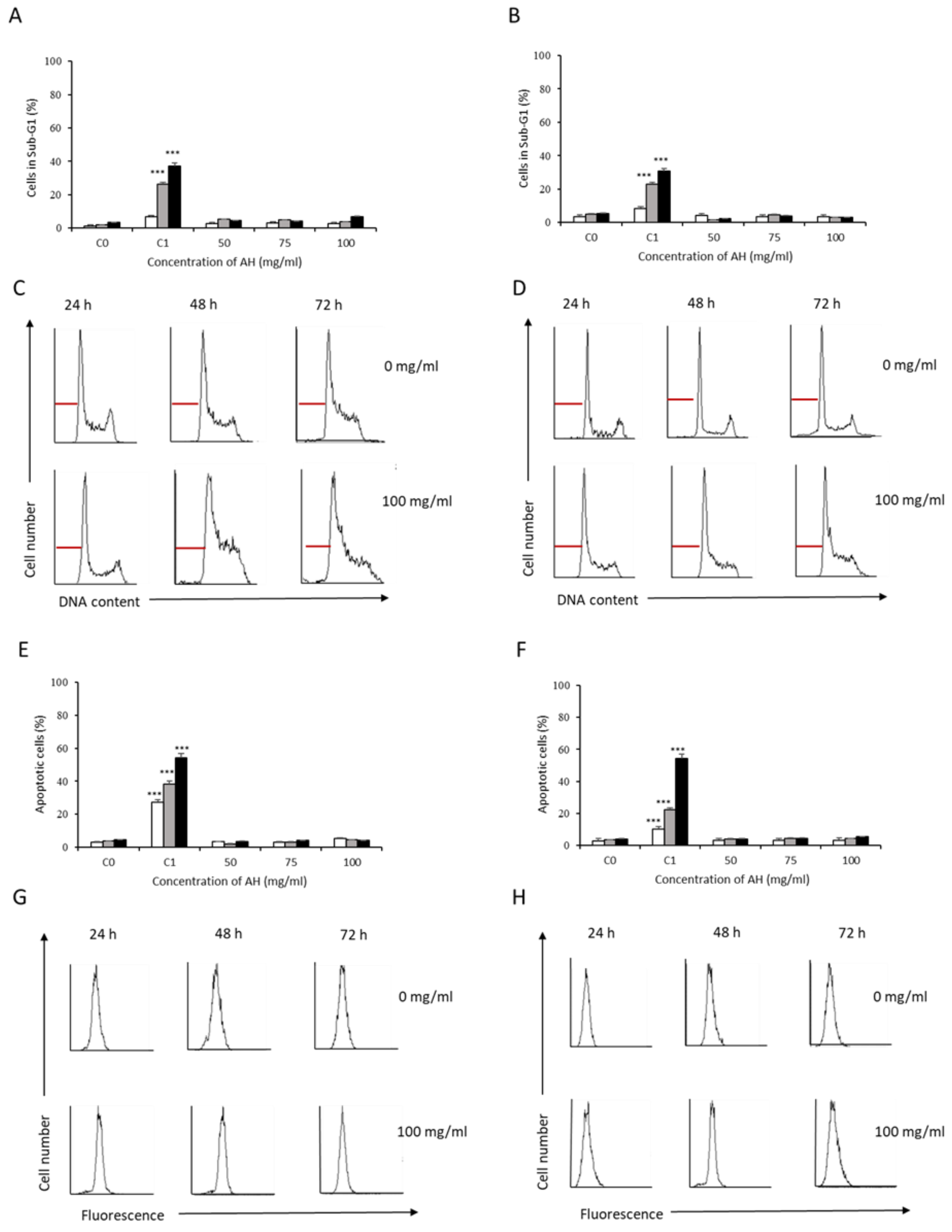

Figure S2. Effect of sample 17 (AH) on human liver cells apoptosis. The cell cycle was determined by flow cytometry. HepG2 (A) and WRL-68 (B) cells were treated with different doses of sample 17 (50, 75, and 100 mg/ml) for 24 (□), 48 (■), and 72 hours (■). Representative histograms of the cell cycle of HepG2 (C) and WRL-68 (D) cells are shown; the red line indicates Sub-G1 phase. The percentage of apoptosis was analyzed by flow cytometry using the TUNEL assay. HepG2 (E) and WRL-68 (F) cells were cultured with different doses of sample 17 (50, 75, and 100 mg/ml) for 24 (□), 48 (■), and 72 hours (■). Representative histograms of the fluorescence intensity in HepG2 (G) and WRL-68 (H) cells are presented. C_0, untreated cells; C_1, cells treated with etoposide (50 μM). Asterisks indicate a significant difference from the control (C_0). ***$P \leq 0.001$.

Table S1. Effect of samples 2, 3, 4, 7, 8, 9, 11 and 13 (100 mg/mL at 72 h) on human liver cells apoptosis analyzed by flow cytometry using the TUNEL assay.

Sample	Code	Apoptotic cells (%) HepG2	Apoptotic cells (%) WRL-68
2	TH+2RJ	8.28 ± 1.22	3.24 ± 2.73
3	TH+10RJ	8.53 ± 0.79	6.67 ± 1.94
4	TH+2PR	6.61 ± 2.30	5.49 ± 1.40
7	CH+2RJ	4.19 ± 1.23	6.11 ± 0.86
8	CH+10RJ	5.71 ± 2.34	6.13 ± 1.03
9	CH+2PR	5.95 ± 1.92	8.91 ± 0.67
11	TH+2RJ+2PR	4.74 ± 2.11	8.08 ± 1.12
13	CH+2RJ+2PR	4.13 ± 1.68	6.12 ± 2.45

Data are expressed as the means ± standard deviation (n = 3).
